# Supplementary material for: One Scaffold, Two Conformations: The Ring-Flip of the Messenger InsP8 Occurs under Cytosolic Conditions
Source: Biomolecules. 2023 Apr 4;13(4):645. doi: 10.3390/biom13040645 (PMC10135964; doi:10.3390/biom13040645)
Supplement: Supplementary file 1 [file biomolecules-13-00645-s001.zip › biomolecules-2283249-supplementary.pdf]

## 1. Supplementary Figures

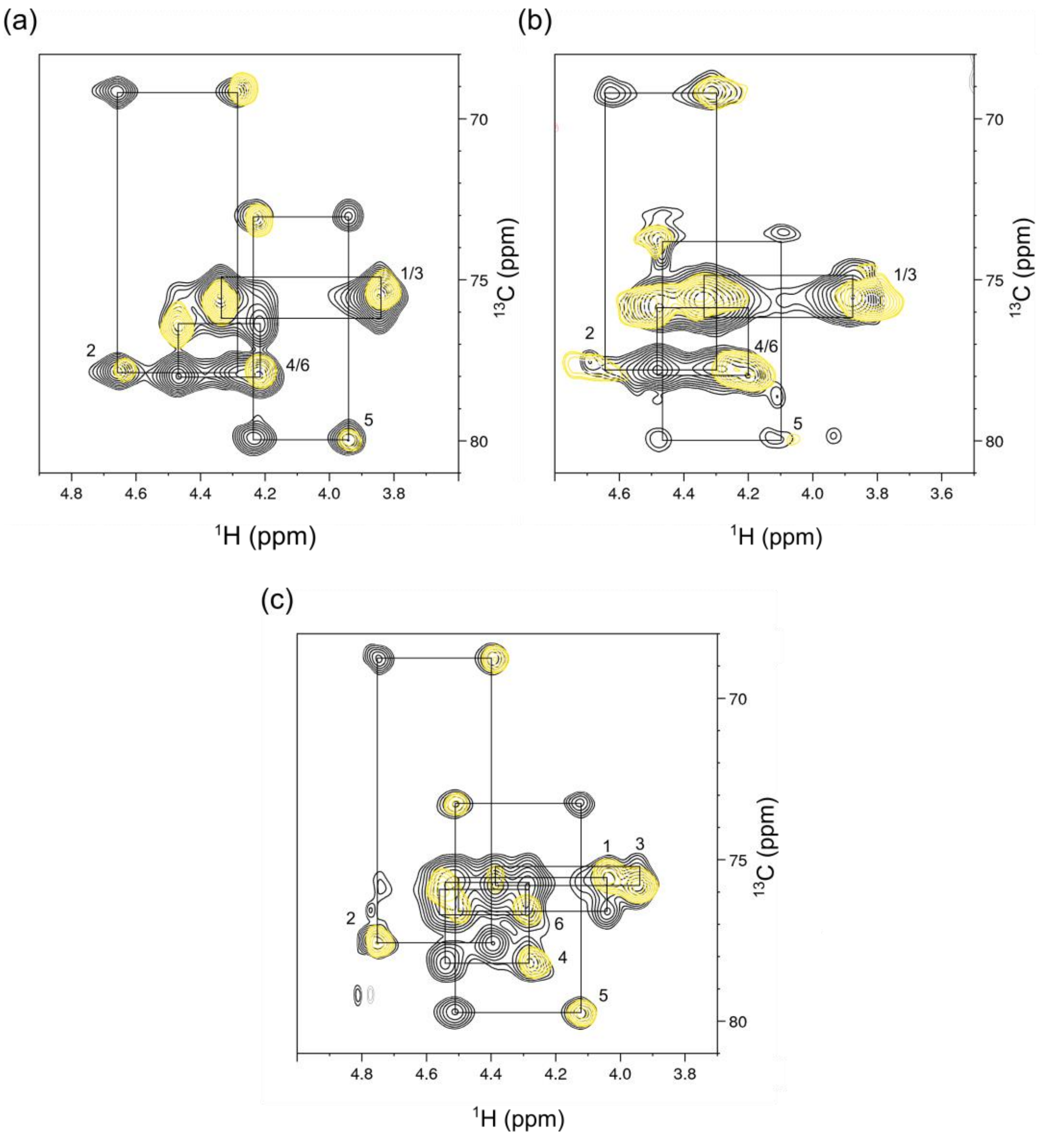


**Figure S1.** Assignment of HMQC spectra in axial conformation (**a**) $InsP_6$, (**b**) 5PP-$InsP_5$ and (**c**) $InsP_8$. Yellow: BIRD-HMQC NMR spectra. Black: NOESY-HMQC spectra. The NOESY method creates cross-peaks between corresponding peaks of the same proton in the two conformations.

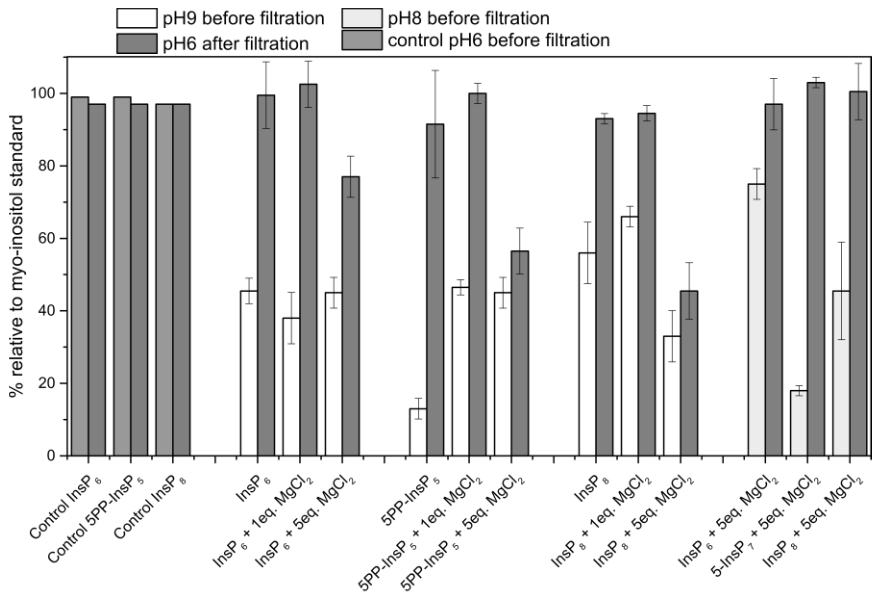


**Figure S2.** Precipitation tests with 50µM $InsP_6$, 5PP-$InsP_5$ and $InsP_8$ in the presence of 0/1/5 equivalents $MgCl_2$, 150 mM KCl and 10 mM NaCl at pH* 9, controls at pH* 6. BIRD-HMQC NMR spectra were recorded, and peak intensity was quantified relative to a myo-inositol standard, which cannot form complexes and therefore does not precipitate with $Mg^{2+}$. Samples were incubated over night at 4 °C, then putative precipitates were removed by filtration through a 0.22 µm RC syringe filter, samples brought to pH* 6 and measured again. If full intensity was recovered, i.e., intensity at pH 6 after filtration was the same as in a control sample at pH 6, it was assumed there was no precipitation in the sample, and reduced intensity at pH* 9 must be due to chemical exchange. Error bars represent SD of two replicates.

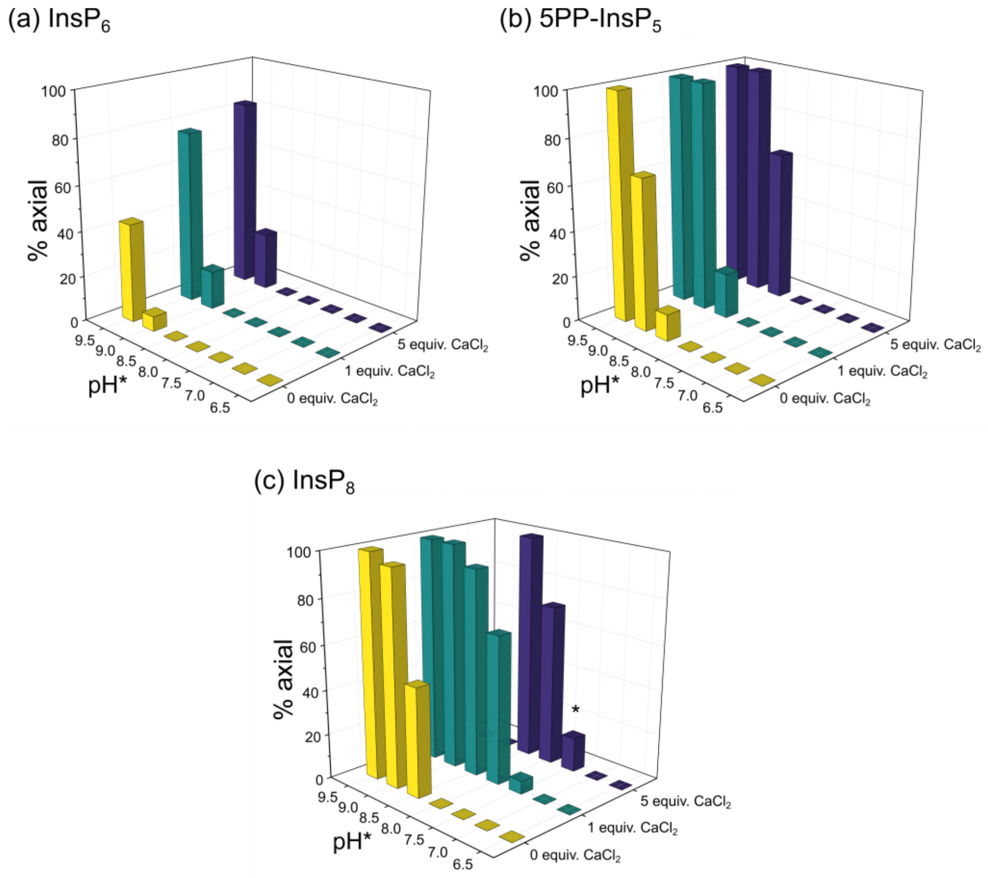


**Figure S3.** Relative abundance of (a) $InsP_6$, (b) 5PP-$InsP_5$ and (c) $InsP_8$ in axial conformation at different pH in the presence of 0/1/5 equivalents $CaCl_2$ and near physiological background of 130 mM KCl and 10 mM NaCl. $^{1}H$,$^{13}C$-HMQC-NMR spectra were integrated to obtain the ratio of eq. to ax.

conformation. The proportion of ax. InsPs increases with pH, $Mg^{2+}$ concentration and phosphorylation state ($InsP_6$ < 5PP-$InsP_5$ < $InsP_8$). Above pH* 8.0, no spectra of $InsP_8$ could be obtained, likely due to a combination of precipitation and peak broadening.

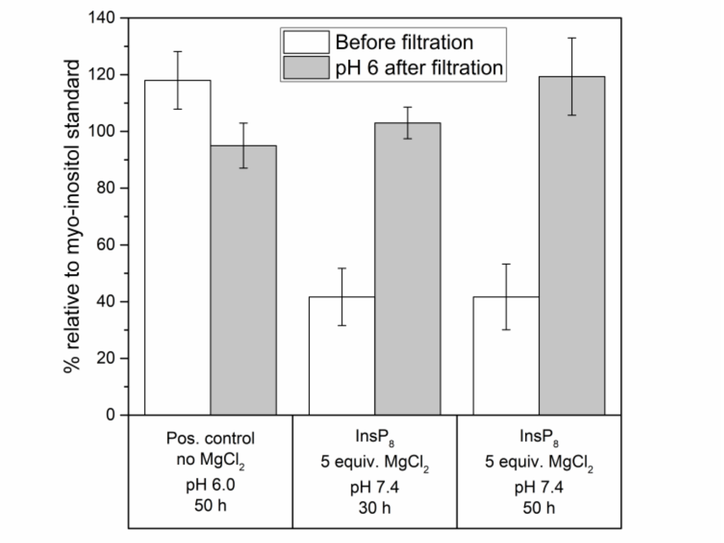


**Figure S4.** Precipitation test under conditions chosen for van't Hoff analysis (pH 7.4, 5 equiv. $MgCl_2$). $InsP_8$ stays in solution during the 50 h required for the experiment. Error bars: SD of three replicates.

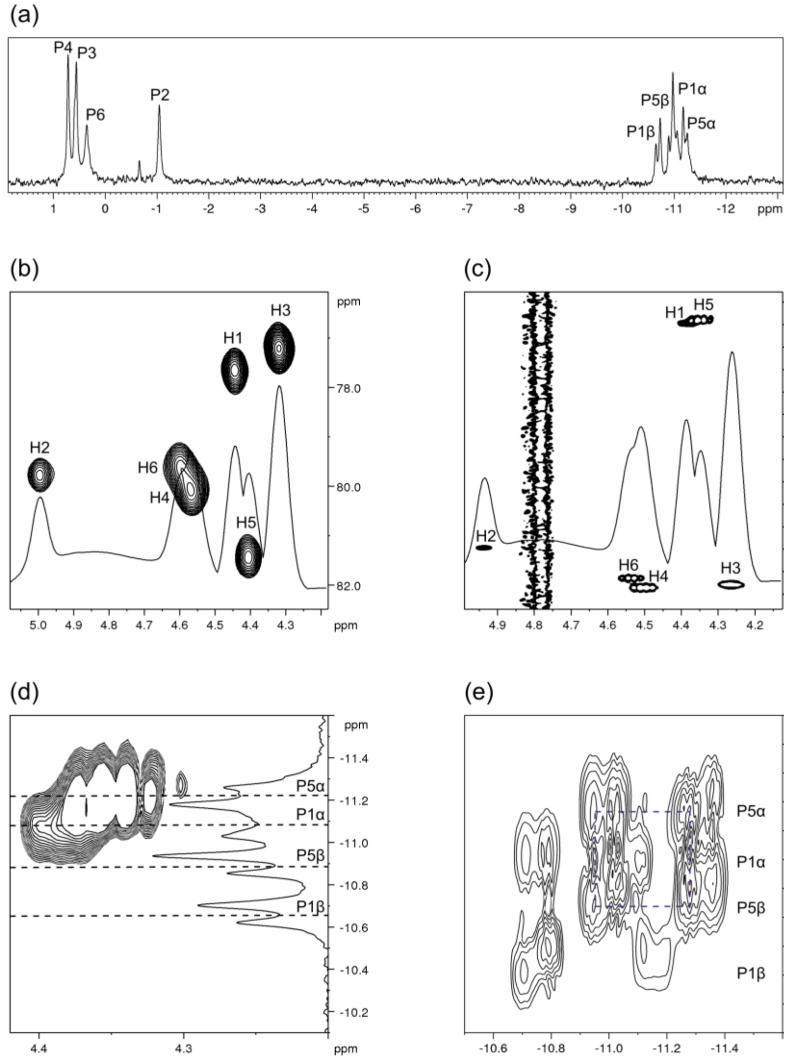

**Figure S5.** Assignment of $^{31}P$–NMR spectrum of $InsP_8$ without coordinating counterions, pH 3. (**a**) 1D $^{31}P$ spectrum. (**b**) $^{1}H,^{13}C$–HMQC and 1D projection. (**c**) $^{1}H,^{31}P$–HMBC + 1D projection of (**b**). (**d**) Zoom on pyrophosphate peaks of HMBC (P1$\alpha$, P5$\alpha$) + pyrophosphate region of 1D $^{31}P$ spectrum. (**e**) $^{31}P,^{31}P$–COSY, zoom on pyrophosphate region.

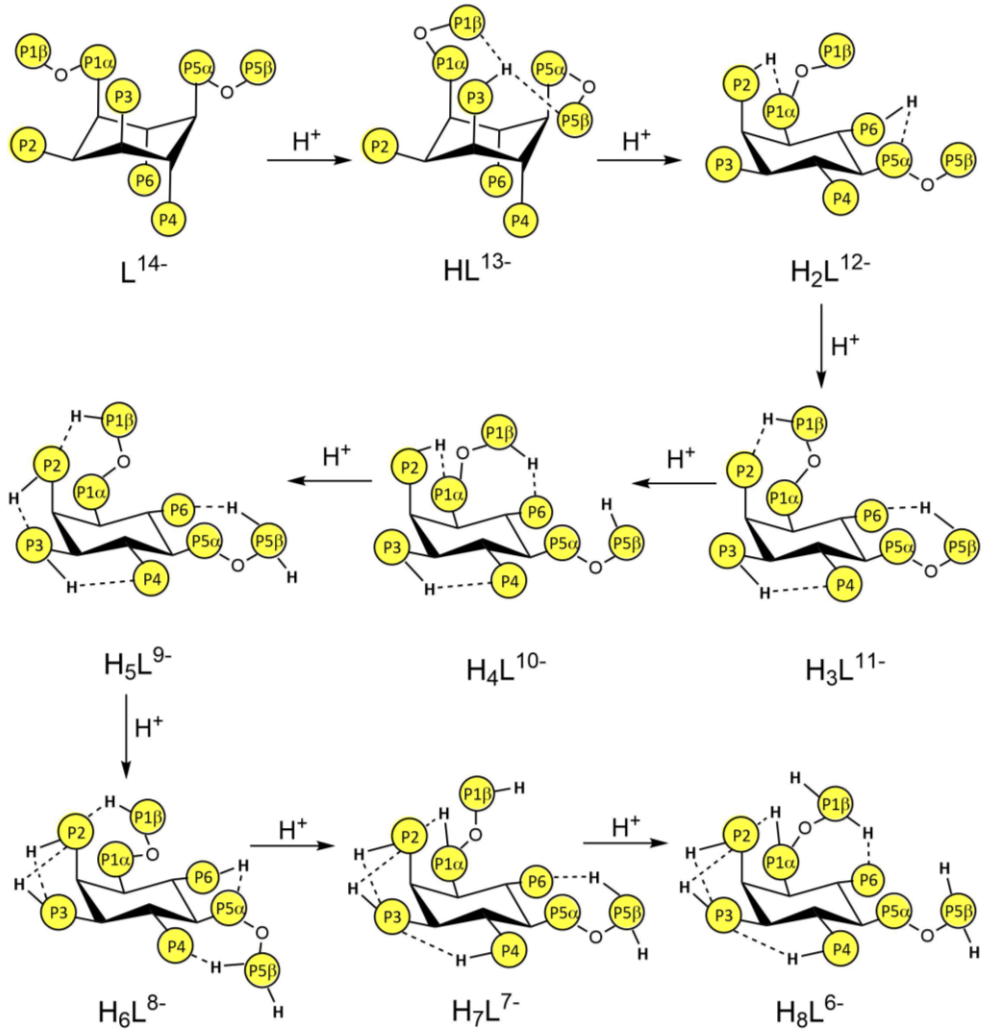


**Figure S6.** Scheme of the most probable protonation patterns for the $InsP_8$ species in the absence of metal ions, as provided by the $^{31}P$ NMR data (I = 0.15 M $NMe_4Cl$, T = 22.0 °C).

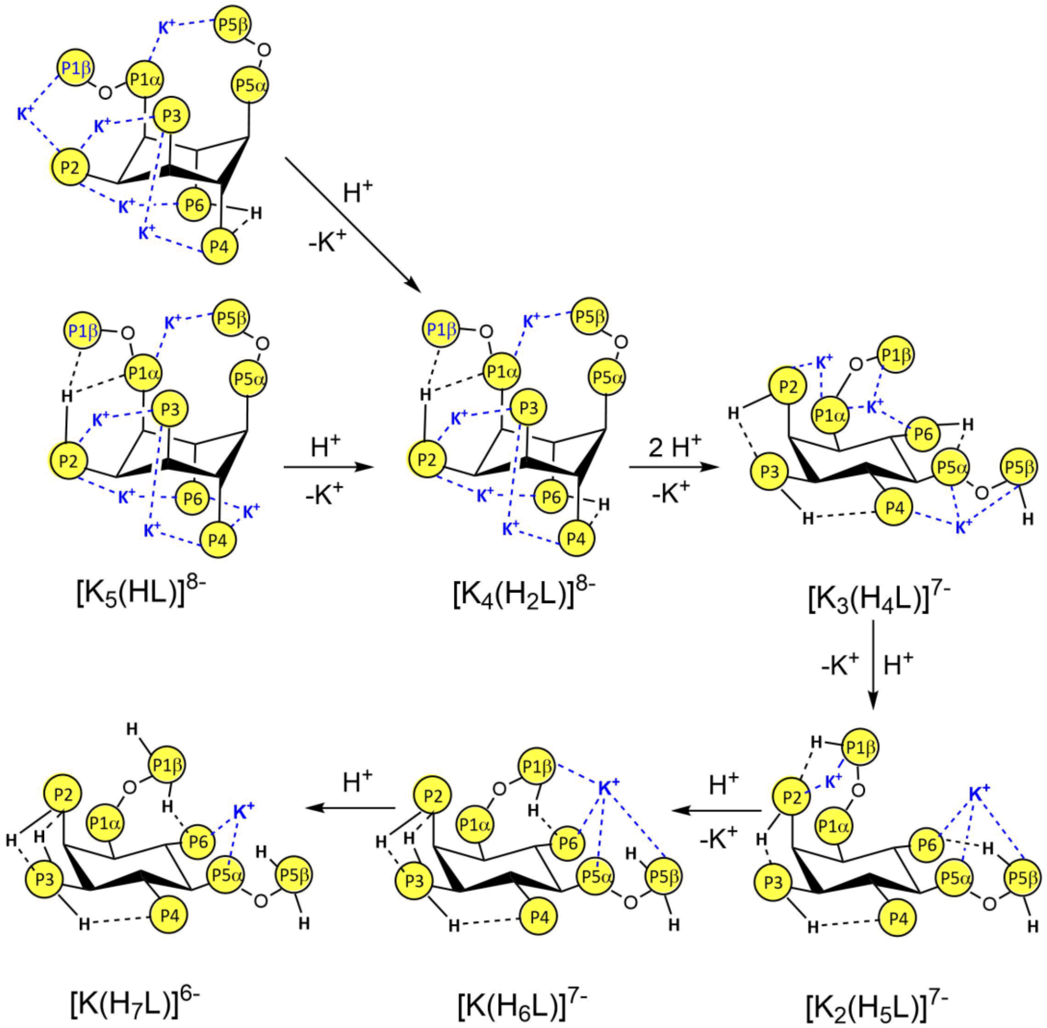


**Figure S7.** Scheme of the most probable protonation and complexation patterns for $K^+$–$InsP_8$ complexes, as provided by the $^{31}P$ NMR data (I = 0.15 M, T = 22.0 °C).

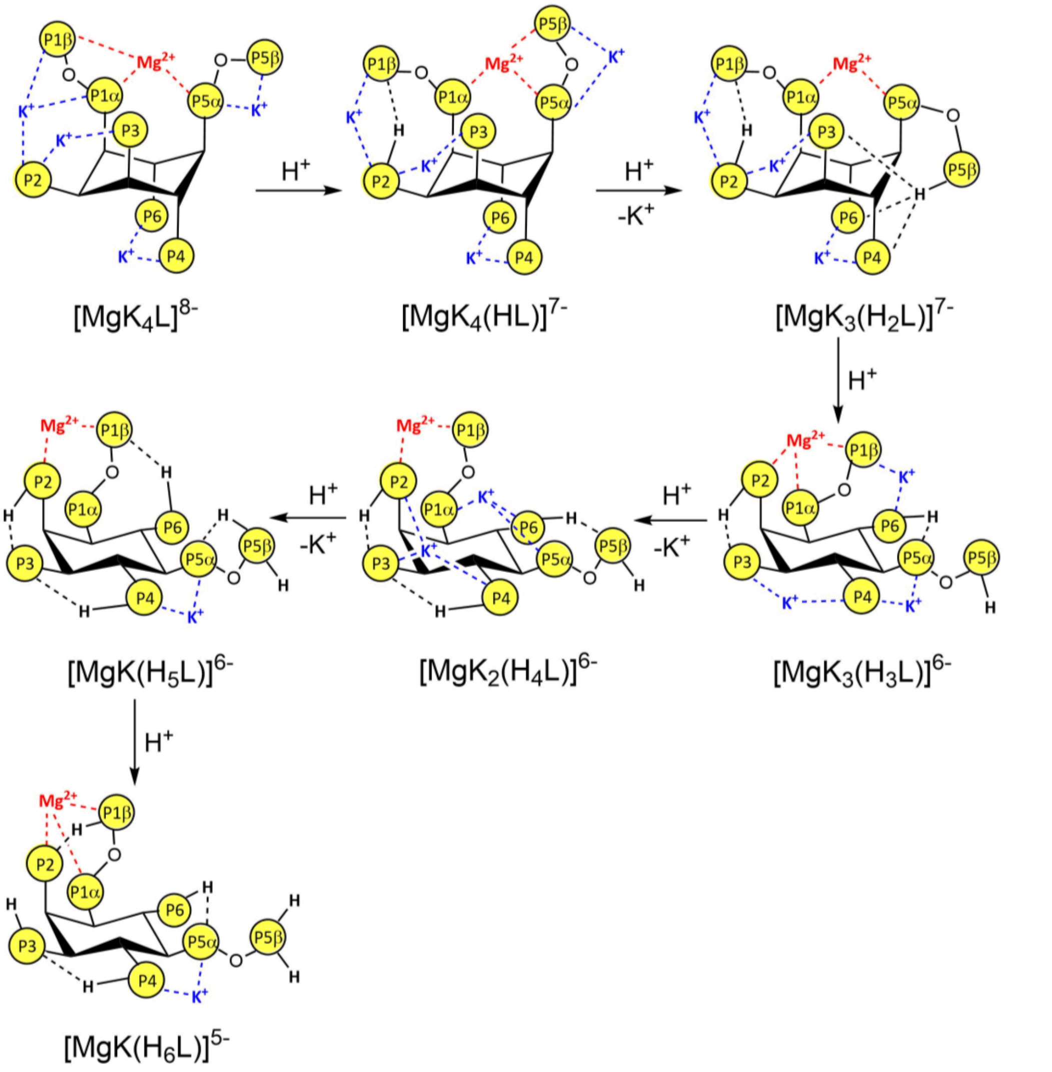


**Figure S8.** Scheme of the most probable protonation and complexation patterns for $Mg^{2+}$-$K^+$–$InsP_8$ complexes, as provided by the $^{31}P$ NMR data (I = 0.15 M, T = 22.0 °C).

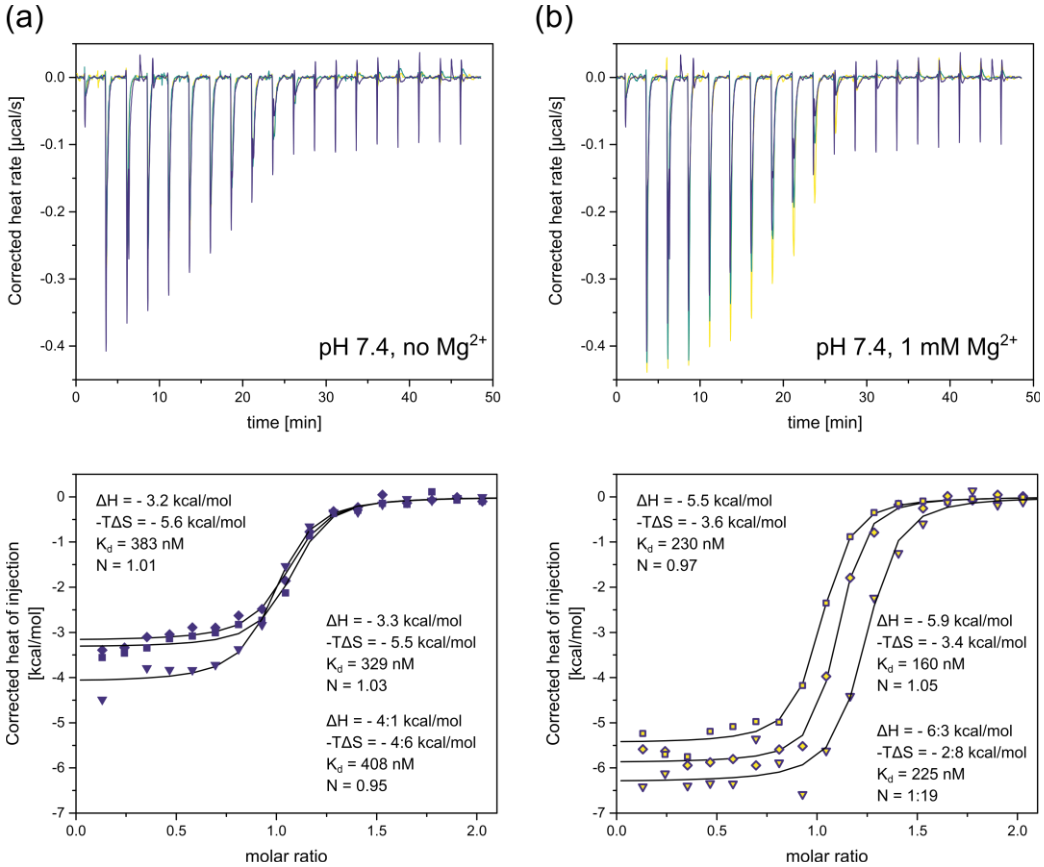


**Figure S9.** Isothermal titration calorimetry of (**a**) $InsP_8$ binding to the yeast VTC2 SPX−domain (50 μM) in ITC binding buffer (25 mM HEPES pH 7.4, 150 mM KCl, 40 mM NaCl, 0.5 mM TCEP) at 25 °C. (**b**) Addition of 1 mM $MgCl_2$ substantially decreases ΔH, ΔS and the binding constant $K_d$.

## 2. Detailed Description of Protonation and Complexation Analysis

### *2.1. Protonation Equilibria and Conformational Change*

The $^{31}P$ NMR profile of $InsP_8$ has eight signals, ascribed to each one of the phosphorous atoms: P1$\alpha$, P1β, P2, P3, P4, P5$\alpha$, P5β and P6. In the absence of metal ions, the $^{31}P$ NMR peaks move downfield as the pH increases, an effect that has already been mechanoquantically described [58].

The fitting of the NMR titration curves (Figure 5a) was carried out using the HypNMR software [36]. As a result, the first eight protonation constants of $InsP_8$ (represented here as $L^{14-}$) could be determined (Table 1). Based on this adjusted chemical model, the solid lines in Figure 5a represent the expected calculated trend for the eight $^{31}P$ NMR chemical shifts. They show an excellent fit to the experimental values. In general, the chemical species detected are similar to those reported for IP6 and 5PCP–IP5 [28,46] although most of the protonation constants are larger, since $InsP_8$ is more negatively charged and stabilize higher protonation states. In this regard, it was even possible to characterize the $H_8L^{6-}$ species; a protonation state that was not detected for the other less charged ligands. Figure 5c shows the species distribution diagram under physiological ionic strength and in the absence of metal cations. The predominant species in solution near pH = 7.4 is $H_5L^{9-}$.

Table A1 lists the calculated individual $^{31}P$ NMR chemical shifts for each phosphorus atom in each of the $InsP_8$ protonated forms. Taking into account that the peaks move upfield upon protonation [58], the order in which each phosphate group is protonated can be determined by computing the change of the individual chemical shifts while $InsP_8$ is successively protonated ($\Delta\delta_P$, Table S1). From this data, Figure A6 depicts the proposed structures for the protonated $InsP_8$ species. It is worth pointing out that some deviations in the $\Delta\delta_P$ values can be accounted for by the fact that other $InsP_8$ species, less abundant, are also present in the system.

The first protonation step gives rise to a significant decrease in the chemical shifts of P3, P1β and P5β (Table S1). This is consistent with two main microspecies in the axial conformation, in which P3 shares its proton with P1β or P5β (Figure S6). The entering of the second proton causes very large $\Delta\delta_P$ values for most of the phosphate groups, an effect that has been associated with a axial-to-equatorial conformational change of the inositol ring for similar systems [26,28,45]. Indeed, the NMR evidence points to a conformational flip of $InsP_8$ at around pH = 11, where $HL^{13-}$ and $H_2L^{12-}$ coexist in solution (Figure 5c). The impact of the conformational transition on the chemical shifts makes it difficult to interpret $\Delta\delta_P$ during the second protonation. Following the relative magnitude and sign of the $\Delta\delta_P$ values, it could be argued that both protons are mainly attached to P2 and P6, being shared to some extent with P1$\alpha$ (lowest $\Delta\delta_P$ values). In the process $HL^{13-} \rightarrow H_2L^{12-}$, P3, P1β and P5β become deprotonated, accounting for their positive $\Delta\delta_P$. It is feasible that P5$\alpha$ is somewhat engaged in an H-bond with P6, since its $\Delta\delta_P$ is much lower than the one for P5β. Interestingly, P4 bears a highly negative $\Delta\delta_P$, probably brought about by the conformational change, which shields the nucleus by forcing P4 to be in an equatorial position, closer to the other ionizable groups.

The $H_2L^{12-} \rightarrow H_3L^{11-}$ reaction is followed by significantly negative $\Delta\delta_P$ values for P3, P1β and P5β, suggesting their protonated state (Table S1). P5$\alpha$ is also shielded, possibly by an inductive effect caused by P5β protonation. The three protons are shared through H-bonds with P2, P4 and P6, all of them exhibiting moderate negative $\Delta\delta_P$ values (see Table S1 and Figure S6). P6 is no longer sharing its proton with P1$\alpha$, giving an explanation for the positive $\Delta\delta_P$ value for the latter. The formation of $H_4L^{10-}$ involves the protonation of P2 ($\Delta\delta_P < 0$), triggering a rearrangement of the H-bonds except for the one linking P3 and P4 (small $|\Delta\delta_P|$ values). P2 shares its $H^+$ with P1$\alpha$ ($\Delta\delta_P < 0$), while P1β does the same with P6 (both with negative $\Delta\delta_P$). In the process, P5β reinforces the covalent bond with the $H^+$, shielding itself and P5$\alpha$.

The fifth protonation step is accompanied by substantially negative $\Delta\delta_P$ values for P1β and P5β (Table S1). The former changes its environment, moving away from P6 and turning to face P2 ($\Delta\delta_P < 0$), with which it shares a proton (Figure S6). Since P2 is already

protonated (but not P6), the P1β-H bond is probably reinforced in the process, causing a partial shielding of the P1β nucleus. On the other hand, P5β becomes protonated, sharing one of the protons with P6 and shielding P5α by an inductive effect (negative $\Delta\delta_P$ values). Although the environment of P4 is almost the same (small $\Delta\delta_P$), P3 is now establishing a new H-bond (gaining a negative $\Delta\delta_P$) and P1α is no longer sharing a proton (exhibiting a positive $\Delta\delta_P$). The sixth protonation of $InsP_8$ does not involve a change in the environment of the pyrophosphate group at C1 (very small $\Delta\delta_P$ values). However, it causes a similar decrease in P2 and P3 chemical shifts. It is feasible that both phosphate groups form a strong double H-bond, giving rise to negative and similar $\Delta\delta_P$ values. This triggers the movement of P4 towards P5β, sharing a proton with it ($\Delta\delta_P$ for P5β < 0; $\Delta\delta_P$ for P4 > 0 and small). This structural rearrangement leaves a negatively charged zone between P6 and P5α, where the sixth proton is retained ($\Delta\delta_P < 0$ for both nuclei).

A similar structural analysis for $H_7L^{7-}$ and $H_8L^{6-}$ is not straightforward, since most of the NMR signals are affected similarly and the number of possible intramolecular H-bond combinations is considerable. A reasonable assumption would be a continuous and dynamic rearrangement of the protons all over the ligand. Nevertheless, we can guess possible structures for the most abundant microspecies of $H_7L^{7-}$ and $H_8L^{6-}$ based on the corresponding $\Delta\delta_P$ values (Table S1, Figure S6). The seventh proton could be bound mainly to P1α (negative $\Delta\delta_P$ value), promoting a spatial rearrangement of the phosphate groups. P1β is inductively shielded by P1α protonation and suffers an extra upfield effect due to the fact that it stops sharing its $H^+$ with P2. P4 is now back to sharing its proton with P3 (lowering its chemical shift), while P6 is now interacting with P5β. It is possible that, in some of the microspecies, the proton on P1β is shared with P6 through an H-bond, allowing P5β to reinforce the bonds with the two $H^+$ ions. This would account for the negative $\Delta\delta_P$ values registered for P5α, P5β and P6. Finally, the eighth proton is predicted to be linked mainly to P1β (negative $\Delta\delta_P$), being shared with P6. In turn, the pyrophosphate group at C5 moves away from P6, deshielding the latter (positive $\Delta\delta_P$) and retaining both protons ($\Delta\delta_P$ for P5α and P5β < 0).

**Table S1.** Calculated $^{31}$P NMR chemical shifts for InsP$_8$ in the absence and presence of $K^+$ and/or $Mg^{2+}$ ($I$ = 0.15 M, $T$ = 22.0 °C). The change in the chemical shifts due only to the protonation ($\Delta\delta_p$) or complexation ($\Delta\delta_c$) processes are included.

| Species | Calculated Chemical Shifts (ppm) | | | | | | | | $\Delta\delta_p$ (ppm) | | | | | | | | $\Delta\delta_c$ (ppm) [a] | | | | | | | |
|---|---|---|---|---|---|---|---|---|---|---|---|---|---|---|---|---|---|---|---|---|---|---|---|---|
| | P1α | P1β | P2 | P3 | P4 | P5α | P5β | P6 | P1α | P1β | P2 | P3 | P4 | P5α | P5β | P6 | P1α | P1β | P2 | P3 | P4 | P5α | P5β | P6 |
| $L^{14-}$ | −9.877 | −4.293 | 2.624 | 4.412 | 2.656 | −8.786 | −4.378 | 2.482 | --- | --- | --- | --- | --- | --- | --- | --- | --- | --- | --- | --- | --- | --- | --- | --- |
| $HL^{13-}$ | −6.955 | −7.206 | 7.342 | −1.990 | 5.531 | −8.772 | −8.177 | 7.495 | 2.922 | −2.914 | 4.719 | −6.402 | 2.874 | 0.014 | −3.799 | 5.013 | --- | --- | --- | --- | --- | --- | --- | --- |
| $H_2L^{12-}$ | −11.830 | −3.273 | 1.565 | 7.267 | 1.166 | −8.869 | −3.371 | 1.255 | −4.875 | 3.933 | −5.777 | 9.258 | −4.365 | −0.098 | 4.806 | −6.240 | --- | --- | --- | --- | --- | --- | --- | --- |
| $H_3L^{11-}$ | −9.134 | −6.028 | 1.177 | 2.044 | 0.850 | −9.499 | −6.121 | 0.998 | 2.695 | −2.755 | −0.388 | −5.223 | −0.316 | −0.629 | −2.750 | −0.257 | --- | --- | --- | --- | --- | --- | --- | --- |
| $H_4L^{10-}$ | −10.629 | −7.488 | 0.154 | 1.968 | 0.622 | −10.191 | −7.871 | 0.430 | −1.495 | −1.460 | −1.023 | −0.076 | −0.228 | −0.692 | −1.749 | −0.567 | --- | --- | --- | --- | --- | --- | --- | --- |
| $H_5L^{9-}$ | −10.424 | −9.519 | −0.644 | 1.450 | 0.728 | −10.506 | −9.530 | 0.144 | 0.205 | −2.031 | −0.798 | −0.518 | 0.107 | −0.315 | −1.660 | −0.287 | --- | --- | --- | --- | --- | --- | --- | --- |
| $H_6L^{8-}$ | −10.410 | −9.470 | −1.062 | 0.872 | 0.885 | −10.762 | −10.046 | −0.139 | 0.014 | 0.048 | −0.418 | −0.579 | 0.157 | −0.256 | −0.516 | −0.282 | --- | --- | --- | --- | --- | --- | --- | --- |
| $H_7L^{7-}$ | −10.988 | −10.250 | −1.063 | 0.610 | 0.810 | −10.987 | −10.367 | −0.270 | −0.578 | −0.779 | −0.001 | −0.262 | −0.075 | −0.225 | −0.321 | −0.132 | --- | --- | --- | --- | --- | --- | --- | --- |
| $H_8L^{6-}$ | −11.040 | −10.672 | −1.075 | 0.552 | 0.703 | −11.220 | −10.910 | 0.262 | −0.052 | −0.423 | −0.012 | −0.058 | −0.107 | −0.233 | −0.543 | 0.532 | --- | --- | --- | --- | --- | --- | --- | --- |
| $[K_5(HL)]^{8-}$ | −8.908 | −4.289 | 3.918 | 4.846 | 3.757 | −8.746 | −4.005 | 3.886 | --- | --- | --- | --- | --- | --- | --- | --- | −1.953 | 2.918 | −3.425 | 6.836 | −1.774 | 0.025 | 4.172 | −3.609 |
| $[K_4(H_2L)]^{8-}$ | −10.271 | −5.910 | 2.984 | 4.064 | 1.898 | −9.210 | −5.015 | 2.143 | −1.363 | −1.621 | −0.934 | −0.782 | −1.859 | −0.464 | −1.011 | −1.743 | 1.559 | −2.636 | 1.419 | −3.203 | 0.732 | −0.341 | −1.644 | 0.888 |
| $[K_3(H_4L)]^{7-}$ | −9.420 | −5.763 | 1.627 | 2.245 | 1.243 | −9.331 | −5.974 | 1.223 | 0.851 | 0.147 | −1.358 | −1.819 | −0.655 | −0.120 | −0.958 | −0.921 | 1.209 | 1.725 | 1.473 | 0.277 | 0.621 | 0.860 | 1.897 | 0.792 |
| $[K_2(H_5L)]^{7-}$ | −10.186 | −8.128 | 0.343 | 1.792 | 1.041 | −9.694 | −7.954 | 0.918 | −0.766 | −2.365 | −1.283 | −0.453 | −0.202 | −0.364 | −1.981 | −0.305 | 0.238 | 1.391 | 0.987 | 0.342 | 0.313 | 0.811 | 1.576 | 0.774 |
| $[K(H_6L)]^{7-}$ | −10.374 | −8.827 | −0.726 | 1.101 | 0.851 | −10.188 | −9.127 | 0.203 | −0.188 | −0.700 | −1.069 | −0.692 | −0.190 | −0.494 | −1.173 | −0.715 | 0.036 | 0.643 | 0.337 | 0.229 | −0.035 | 0.574 | 0.919 | 0.342 |
| $[K(H_7L)]^{6-}$ | −10.827 | −10.002 | −0.669 | 0.640 | 0.870 | −10.457 | −10.040 | 0.247 | −0.452 | −1.175 | 0.056 | −0.461 | 0.020 | −0.269 | −0.913 | 0.044 | 0.161 | 0.247 | 0.394 | 0.030 | 0.060 | 0.530 | 0.328 | 0.517 |
| | | | | | | | | | | | | | | | | | | | | | | | | |
| $[MgK_4L]^{8-}$ | −9.242 | −3.961 | 3.880 | 4.923 | 3.520 | −8.918 | −3.963 | 3.641 | --- | --- | --- | --- | --- | --- | --- | --- | −0.334 | 0.328 | −0.038 | 0.077 | −0.236 | −0.172 | 0.042 | −0.245 |
| $[MgK_4(HL)]^{7-}$ | −9.748 | −4.215 | 3.451 | 4.608 | 3.165 | −9.596 | −4.204 | 3.495 | −0.506 | −0.254 | −0.429 | −0.315 | −0.355 | −0.677 | −0.242 | −0.146 | −0.840 | 0.073 | −0.467 | −0.238 | −0.592 | −0.850 | −0.200 | −0.391 |
| $[MgK_3(H_2L)]^{7-}$ | −10.156 | −4.755 | 3.442 | 2.763 | 0.948 | −9.647 | −5.995 | 1.490 | −0.408 | −0.540 | −0.009 | −1.845 | −2.217 | −0.051 | −1.790 | −2.005 | 0.115 | 1.155 | 0.457 | −1.302 | −0.950 | −0.437 | −0.980 | −0.653 |

| | | | | | | | | | | | | | | | | | | | | | | | | |
|---|---|---|---|---|---|---|---|---|---|---|---|---|---|---|---|---|---|---|---|---|---|---|---|---|
| $[MgK_3(H_3L)]^{6-}$ | −10.855 | −6.080 | −0.706 | 1.920 | 1.185 | −9.906 | −6.671 | 0.754 | −0.698 | −1.325 | −4.147 | −0.843 | 0.237 | −0.259 | −0.676 | −0.736 | −1.720 | −0.052 | −1.883 | −0.124 | 0.335 | −0.408 | −0.549 | −0.244 |
| $[MgK_2(H_4L)]^{6-}$ | −8.919 | −6.485 | 0.514 | 2.342 | 0.780 | −9.453 | −6.962 | 0.852 | 1.936 | −0.405 | 1.220 | 0.422 | −0.405 | 0.454 | −0.292 | 0.098 | 0.501 | −0.722 | −1.112 | 0.097 | −0.463 | −0.122 | −0.989 | −0.370 |
| $[MgK(H_5L)]^{6-}$ | −10.258 | −7.811 | −1.014 | 1.306 | 0.683 | −10.202 | −8.365 | 0.023 | −1.339 | −1.326 | −1.528 | −1.036 | −0.097 | −0.749 | −1.403 | −0.830 | −0.072 | 0.316 | −1.357 | −0.486 | −0.359 | −0.507 | −0.411 | −0.895 |
| $[MgK(H_6L)]^{5-}$ | −11.260 | −10.039 | −1.000 | 0.557 | 0.597 | −10.874 | −10.079 | −0.180 | −1.002 | −2.228 | 0.015 | −0.749 | −0.086 | −0.672 | −1.714 | −0.202 | −0.886 | −1.212 | −0.274 | −0.544 | −0.254 | −0.686 | −0.952 | −0.382 |

[a] $\Delta\delta_c$ values for each $Mg^{2+}$ complex are calculated taking the equally protonated $K^+$ species as a reference. For $[MgK_4L]^{8-}$, $\Delta\delta_c$ was calculated taking $[K_5(HL)]^{8-}$ as a reference. For $[MgK_3(H_3L)]^{6-}$, $\Delta\delta_c$ was calculated taking $H_3L^{11-}$ as a reference. Chemical and structural behavior under physiological levels of $K^+$.

The $^{31}P$ NMR signals of $InsP_8$ in the presence of an intracellular concentration of $K^+$ vary with pH as shown in Figure 5. When comparing with the same titration curves for $InsP_8$, it is clear that all peaks suffer a downfield effect above pH 3, where $K^+$ ions are present in excess. This phenomenon suggests the formation of stable $K^+$-$InsP_8$ adducts bearing different protonation states. According to previous reports [26,28,45], two main phenomena modulate the behavior of the phosphorus chemical shifts with pH: a deshielding effect brought about by $K^+$ complexation, and a shielding effect upon each protonation step.

Figure 5b depicts the mathematical fitting of the titration curves. The model satisfactorily explains the experimental trends, and allows to unveil the chemical speciation of the system. As shown in Table 1, six $K^+$ complexes were detected: $[K_5(HL)]^{8-}$, $[K_4(H_2L)]^{8-}$, $[K_3(H_4L)]^{7-}$, $[K_2(H_5L)]^{7-}$, $[K(H_6L)]^{7-}$ and $[K(H_7L)]^{6-}$, being all of them thermodynamically very stable considering the low ionic potential of potassium ions. Taking into account the high charge displayed by $InsP_8$ anionic species, it is not surprising that the $K^+$- $InsP_8$ adducts have this remarkable stability, based on strong electrostatic interactions. In fact, their equilibrium formation constants are much higher than those reported for 5PCP-IP5 and IP6 potassium complexes (see Table 1). Figure 5d shows the species distribution of $InsP_8$ in the presence of physiological amounts of $K^+$ ions. The complexation processes are operative even at pH values as low as 2, giving rise to polynuclear species that are progressively less protonated and with higher nuclearity as the pH increases. The predominant species in solution at physiological pH is $[K_2(H_5L)]^{7-}$.

To unveil the inframolecular details of the $K^+$–containing species, the change in the calculated individual chemical shifts during the protonation ($[K_x(H_yL)] + m\ H^+ \rightarrow [K_{x'}(H_{y+m}L)]$; $\Delta\delta_P$) and complexation ($H_yL + x\ K^+ \rightarrow [K_x(H_yL)]$; $\Delta\delta_C$) processes was analyzed (see Table A1). With that information, we could track the location of the $H^+$ and $K^+$ ions in the most abundant complexes (Figure A7). In any case, it is worth noting that other less important microspecies may be present in the system, contributing to the values observed for $\Delta\delta_P$ and $\Delta\delta_C$.

Consistent with what was reported for phytate [26] and 5PCP-IP5 [28], it was assumed that the conformational state of $[K_5(HL)]^{8-}$ was axial. According to Table S1, this species displays higher $\Delta\delta_C$ values for P3, P5β and P1β, indicating that they are expected to be deprotonated (they share a proton in $HL^{13-}$; see Figure S6) and linked to $K^+$ ions after the step $HL^{13-} + 5\ K^+ \rightarrow [K_5(HL)]^{8-}$. Meanwhile, P1$\alpha$, P2, P4 and P6 have negative $\Delta\delta_C$ values, possibly sharing a proton through intramolecular H-bonds. This implies the coexistence of two main axial complexes, with the only proton located between P4 and P6, or between P1$\alpha$ and P2. The latter $H^+$ may also be shared with P1β to some extent, giving an explanation for the fact that the $\Delta\delta_C$ value for this nucleus is smaller than the one for P3 and P5β.

With a decrease in the pH, the species $[K_5(HL)]^{7-}$ becomes protonated and releases one $K^+$ ion, giving rise to $[K_4(H_2L)]^{7-}$. According to the $\Delta\delta_P$ values, during this reaction the two protons are stabilized in the same location found in the two main complexes of $[K_5(HL)]^{7-}$ (Figure S7): P4/P6 and P2/P1$\alpha$/P1β (lowest $\Delta\delta_P$ values). This structural alteration triggers the release of the $K^+$ ion initially located between P2 and P1β in one of the $[K_5(HL)]^{7-}$ microspecies, or that linked to P4 and P6 in the other complex. In spite of it, all of the $\Delta\delta_P$ values are negative, suggesting that there may be other minor microspecies where P3, P5$\alpha$ and P5β are bound to protons. The $\Delta\delta_C$ values for $[K_4(H_2L)]^{7-}$ are difficult to interpret, since in the process $H_2L^{12-}$ (1a5e) $\rightarrow [K_4(H_2L)]^{7-}$ (axial) there is a conformational flip involved. Indeed, P1$\alpha$, P2, P4 and P6 suffer a downfield effect, while P1β, P3, P5$\alpha$ and P5β are shielded, in line with an axial-to-equatorial transition (see $\Delta\delta_P$ values for $H_2L^{12-}$ in Table S1).

When the pH is further lowered, one potassium ion is exchanged by two $H^+$, producing $[K_3(H_4L)]^{7-}$. The absence of a triprotonated $K^+$ complex is remarkable. It is likely that a structural change promotes the stabilization of four (instead of three) $H^+$ in the complex. A feasible explanation is based on an axial-to-equatorial conformational flip, which allows

the ionizable groups to be closer, stabilizing a higher number of $H^+$ through intramolecular H-bonds. The NMR evidence supports this assumption, indicating the coexistence of both conformational states at around pH = 8.5–9, where $[K_4(H_2L)]^{7-}$ and $[K_3(H_4L)]^{7-}$ are abundant in solution (Figures 3 and 5d). As it was reported for $InsP_6$ and 5PCP-$InsP_5$ [26,28], the axial conformation is the most thermodynamically stable for low protonated $K^+$ complexes, providing a preformed structure with negatively charged cavities capable of binding the alkaline ions, while reducing the ring distortion. When the $H^+$ concentration is high enough, the protonation and formation of strong intramolecular H-bonds force the majority of the ionizable groups to be closer, in equatorial positions. Returning to $[K_3(H_4L)]^{7-}$, the information in Table S1 suggests that the four protons are linked to P2, P3, P5β and P6, since they exhibit the lowest $\Delta\delta_P$ values. A part of those $H^+$ are shared to some extent with P5$\alpha$ and P4, which explains their smaller but negative $\Delta\delta_P$. According to the $\Delta\delta_c$ values, the potassium ions are closer to P1$\alpha$, P1β, P2, P5$\alpha$ and P5β. P4 and P6 have positive but smaller $\Delta\delta_c$, also participating to some extent in the coordination scheme (Figure S7).

The next protonation step, $[K_3(H_4L)]^{7-} \rightarrow [K_2(H_5L)]^{7-}$, is accompanied by a an upfield shift of the signals, especially for P1β, P2 and P5β. In this sense, it is likely that the entering proton is shared between P1β and P2, while P5β, already protonated, starts to share a second $H^+$ with P6, inductively shielding P5$\alpha$ (Figure S7). P1$\alpha$ also exhibits a moderately negative $\Delta\delta_P$, probably brought about by the release of the $K^+$ ion bound to it in $[K_3(H_4L)]^{7-}$. As shown in Figure S7, the two $K^+$ ions in $[K_2(H_5L)]^{7-}$ are coordinated by P1β, P2, P5$\alpha$, P5β and P6. Indeed, these nuclei display the largest $\Delta\delta_c$ values, since in the process $H_5L^{9-} \rightarrow [K_2(H_5L)]^{7-}$ they come near both $K^+$ cations (deshielding effect), while the protonation scheme remains unchanged.

Towards acidic media, the mononuclear species become abundant: $[K(H_6L)]^{7-}$ and $[K(H_7L)]^{6-}$ (Figure 5d). From $[K_2(H_5L)]^{7-}$ to $[K(H_6L)]^{7-}$, P2 is protonated, sharing two $H^+$ with P3 and moving away from P1β (Figure S7). This structural change promotes the release of one of the two $K^+$ ions, formerly shared between P2 and P1β, allowing the latter to establish an H-bond with P6 and to make contact, to a certain extent, with the remaining $K^+$ ion. In the whole process, all of the ionizable groups are shielded, being P1β, P2, P3, P5β and P6 the most affected, since they share the six protons (Table S1). P5$\alpha$ is probably shielded by an inductive effect from P5β. P1$\alpha$ and P4, however, seem to be mostly deprotonated and interact with $H^+$ only to a moderate level. Regarding the $\Delta\delta_c$ values, the step $H_6L^{8-} \rightarrow [K(H_6L)]^{7-}$ involves a downfield change in the chemical shifts of P1β, P5$\alpha$, P5β and P6, suggesting that they are somehow near the $K^+$ ion. P2 is also deshielded in this process, but this effect is due to the fact that it is in fact deprotonated upon complexation (see the structure of $H_6L^{8-}$ in Figure S6).

Finally, the protonation of $[K(H_6L)]^{7-}$ generates $[K(H_7L)]^{6-}$, and the NMR signals of P1$\alpha$, P1β, P3 and P5β move upfield (Table S1). Figure A7 shows a possible main microspecies, where P1β links the entering proton (shielding effect for P1β and P1$\alpha$). It is likely that the $K^+$ ion moves away from P5β and P1β ($\Delta\delta_P < 0$). In the case of P3, the structural change associated with the protonation step may alter the intramolecular H-bonds, making the three $H^+$ ions closer to the phosphate group and shielding the nucleus. Starting from $H_7L^{7-}$, the formation of $[K(H_7L)]^{6-}$ entails the deshielding of P5$\alpha$ and P6, indicating that both ionizable groups are near the $K^+$ ion. P1β is also affected but to a much lesser extent, suggesting that in some microspecies it may be somehow involved in the metal coordination scheme. Interestingly, P2 and P5β also have $\Delta\delta_c > 0$. With respect to the former, it is located near three $H^+$ ions in $H_7L^{7-}$, but it is surrounded by only two protons in $[K(H_7L)]^{6-}$. In the case of P5β, the analysis is not straightforward. The observed deshielding effect may be due to the coexistence of minor microspecies in which P5β is part of the $K^+$ coordination polyhedra.

### *2.2. Interaction of $InsP_8$ with $K^+$ and $Mg^{2+}$: Thermodynamic and Structural Characterization*

Figure 6a depicts the $^{31}P$ NMR titration curves for an equimolar solution of $InsP_8$ and $Mg^{2+}$ in the presence of physiological amount of $K^+$ ions. Compared to the chemical shifts

without $K^+$ in Figure 5b, it is evident that the magnesium ion causes an alteration of the phosphorous chemical shifts, which is positive or negative depending on the pH value. This phenomenon gives evidence of the interaction between $InsP_8$ and $Mg^{2+}$ ions, which is expected to be based on the formation of coordinative species.

The experimental titration curves were fitted employing the HypNMR software, testing a wide range of different chemical models. The best result was obtained with the model shown in Table 1, which gives an excellent fitting of the experimental data (Figure 6a). Only mononuclear magnesium coordination complexes were detected, bearing different number of linked $K^+$ ions and protonation states: $[MgK_4L]^{8-}$, $[MgK_4(HL)]^{7-}$, $[MgK_3(H_2L)]^{7-}$, $[MgK_3(H_3L)]^{6-}$, $[MgK_2(H_4L)]^{6-}$, $[MgK(H_5L)]^{6-}$ and $[MgK(H_6L)]^{5-}$. The species distribution diagram under equimolar $Mg^{2+}$: $InsP_8$ conditions, shown in Figure 6b, indicates that these complex species are abundant over the entire pH range tested. This is due to the high stability of the $Mg^{2+}$-$K^+$-$InsP_8$ complexes, which exhibit formation equilibrium constants much larger than the analogous species for 5PCP-$InsP_5$ (see Table 1) [28]. At physiological pH, the most abundant species is $[MgK_3(H_3L)]^{6-}$.

The HypNMR software also provides the individual chemical shifts adjusted for each $^{31}P$ nucleus in every complex species. From them, we could calculate the change in the chemical shifts triggered by $InsP_8$ protonation and complexation processes ($\Delta\delta_P$ and $\Delta\delta_c$). The results are listed in Table S1. To interpret the data, it is worth taking into account that, in the presence of $Mg^{2+}$, the $^{31}P$ chemical shifts are affected positively or negatively depending on two main factors, (i) a downfield effect brought about by the ligand deprotonation and/or association with $K^+$, and (ii) an upfield contribution brought about by $Mg^{2+}$ complexation [45]. Bearing this in mind, and starting from data in Table A1, Figure S8 depicts the most probable protonation and complexation schemes for the seven $Mg^{2+}$-$K^+$-$InsP_8$ complexes. In any case, it is worth noting that other minor complex microspecies with different molecular structures might coexist in solution, contributing to the change registered for the NMR signals as pH varies.

Above pH = 10.5, the predominant species is $[MgK_4L]^{8-}$. According to previous studies on similar systems [26,28], an axial conformation can be safely assumed when the ligand's protonation degree is low. In the process $[K_5(HL)]^{8-} \rightarrow [MgK_4L]^{8-}$, there is an upfield effect on P1$\alpha$ signal, suggesting that it may be involved in magnesium complexation (see the corresponding $\Delta\delta_c$ value). The coordination environment is probably completed with P5$\alpha$ ($\Delta\delta_c < 0$). It is feasible that P1β is also involved in the coordination of the $Mg^{2+}$ ion, but only up to a point, since it shares a potassium ion with P1$\alpha$ and P2. In this regard, it is located farther away from the magnesium ion, suffering a partial deshielding by the divalent charge when going from $[K_5(HL)]^{8-}$ to $[MgK_4L]^{8-}$ (slightly positive $\Delta\delta_c$). P4 and P6 are somewhat shielded upon magnesium complexation, due to the fact that during $[K_5(HL)]^{8-} \rightarrow [MgK_4L]^{8-}$ step, both phosphate groups change from being in contact with one or two potassium ions each to share only one $K^+$ cation. P5β signal is practically not altered upon coordination with magnesium, indicating that it does not participate in the coordination scheme to a great extent, keeping the connection with one $K^+$ ion (see the structure of $[K_5(HL)]^{8-}$). All of the nuclei are in contact with one $K^+$ ion, except P2 that binds two, in line with the fact that its NMR signal is the most deshielded during the process $L^{14-} \rightarrow [MgK_4L]^{8-}$ (compare their chemical shifts in Table S1).

At lower pH values, the protonation of $[MgK_4L]^{8-}$ gives rise to $[MgK_4(HL)]^{7-}$ species (Figure 6b). Data in Table S1 is compatible with the structure shown in Figure S8. P2 is probably protonated, displaying a negative $\Delta\delta_P$ in the process $[MgK_4L]^{8-} \rightarrow [MgK_4(HL)]^{7-}$. This proton is shared with P1β, which in turn moves away from the magnesium ion, leaving the first coordination sphere. This $Mg^{2+}/H^+$ exchange causes a small upfield effect in P1β signal (small negative $\Delta\delta_P$). On the other hand, P1$\alpha$ loses the connection with the potassium ion, reinforcing the coordinative bond with $Mg^{2+}$, giving an explanation for the associated negative $\Delta\delta_P$ value. Interestingly, P5$\alpha$ displays the most negative $\Delta\delta_P$. A possible explanation lies in the reinforcement of its bond with $Mg^{2+}$, probably brought about by a change in the coordination scheme that allows P5β to also participate, to some extent, in the binding of magnesium (P5β $\Delta\delta_P < 0$). This protonation and complexation scheme is

also compatible with the $\Delta\delta_c$ values in Table S1, which quantify the change in the chemical shifts during the process $[K_5(HL)]^{8-} \rightarrow [MgK_4(HL)]^{7-}$. P1$\alpha$ and P5$\alpha$ exhibit the most negative $\Delta\delta_c$ values, since they are involved in the magnesium coordination. P5β suffers a shielding effect, since it is also linked to the $Mg^{2+}$ ion, although the phenomenon is less marked, which points to a partial involvement in the coordination scheme. As expected, P1β signal is practically not influenced upon magnesium complexation, because it is associated with one $K^+$ and one $H^+$ ions in both species, $[K_5(HL)]^{8-}$ and $[MgK_4(HL)]^{7-}$. P2 chemical shift, however, suffers an upfield effect during the binding of $Mg^{2+}$, due to the fact that in the process it consolidates its protonation (in one of the microspecies of $[K_5(HL)]^{8-}$ P2 is deprotonated and connected to 3 $K^+$ cations). In addition, P4 and P6 have negative $\Delta\delta_c$ values, an effect brought about by the decrease in the number of potassium cations near them during the process $[K_5(HL)]^{8-} \rightarrow [MgK_4(HL)]^{7-}$.

With a further decrease in pH, $[MgK_3(H_2L)]^{7-}$ is formed (Figure 10), with a consequent decrease in P3, P4, P5β and P6 chemical shifts (see $\Delta\delta_P$ values in Table S1). Assuming that the magnesium ion keeps its location between P1$\alpha$ and P5$\alpha$ (P5$\alpha$ $\Delta\delta_P$ is almost negligible), it can be proposed the formation of various microspecies where P5β is protonated, sharing the proton above or below the inositol ring with P3 or P4/P6, respectively. The change in the coordination environment makes the $Mg^{2+}$ ion closer to P1$\alpha$ ($\Delta\delta_P < 0$), probably altering the position of P1β relative to the proton it shares with P2 (P1β $\Delta\delta_P < 0$). P2, however, seems not to suffer from a significant change in its surroundings, or the structural modifications brought about by P1β shift are countered by those caused by P3-P5β H-bond ($\Delta\delta_P \approx 0$). The proposed structural model of $[MgK_3(H_2L)]^{7-}$ is also in agreement with the $\Delta\delta_c$ values calculated for the process $[K_4(H_2L)]^{8-} \rightarrow [MgK_3(H_2L)]^{7-}$. In the reaction, P1β binds a potassium ion ($\Delta\delta_c > 0$), P3 becomes protonated ($\Delta\delta_c < 0$), P4 and P6 start to share only one $K^+$ ion ($\Delta\delta_c < 0$), P5$\alpha$ binds the magnesium ion ($\Delta\delta_c < 0$), and P5β loses its connection to a $K^+$ ion upon protonation ($\Delta\delta_c < 0$). The $\Delta\delta_c$ of P2 and P1$\alpha$ are more difficult to interpret. Apart from the fact that other microspecies may be coexisting in solution, a possible explanation involves the approaching of both phosphate groups towards the $K^+$ ion connected to P3 when the latter becomes protonated, accounting for the somewhat positive $\Delta\delta_c$ values for P2 and P1$\alpha$.

The next protonation step produces $[MgK_3(H_3L)]^{6-}$, abundant in the pH range of 7.2 to 8.2 (Figure 6b). Its formation from $[MgK_3(H_2L)]^{7-}$ is accompanied by a very large negative change in P2 chemical shift ($\Delta\delta_P = -4.147$; see Table S1 and Figure 6a). Moreover, the P2 NMR peak becomes extremely broad at pH values between 8.0 and 8.5, preventing its assignment in the NMR spectra. These features have been previously described as indicative of an axial-to-equatorial conformational flip [26,28]. Therefore, we propose that, within the pH range of 8.0–8.5, a $[MgK_3(H_2L)]^{7-}$ (ax.) → $[MgK_3(H_3L)]^{6-}$ (eq.) is operative in solution. According to the species distribution diagram of Figure 6b, both species coexist in that pH interval, and the predicted relative proportion of both complexes (22% axial/78% equatorial at pH = 7.85; 53% axial/47% equatorial at pH = 8.32) is in excellent agreement with the NMR experimental evidence under similar conditions (32% axial/68% equatorial at pH = 7.85 (pH* = 8.0); 45% axial/55% equatorial at pH = 8.32 (pH* = 8.5) at 4 °C with 1equiv. $MgCl_2$, see Figure 3c). The conformational change makes the $\Delta\delta_P$ values of $[MgK_3(H_3L)]^{6-}$ difficult to interpret. However, and assuming that all of the signals are shielded similarly by the conformational flip, the most negatively affected signals are P1β and P2, suggesting that they play a role in the magnesium coordination. It is feasible that P1$\alpha$ keeps binding the $Mg^{2+}$ ion to a certain extent, since it displays a quite negative $\Delta\delta_P$. In the process $[MgK_3(H_2L)]^{7-} \rightarrow [MgK_3(H_3L)]^{6-}$, P5$\alpha$ leaves the magnesium first coordination sphere and it ends up being surrounded by a $K^+$ and $H^+$ ions (small negative $\Delta\delta_P$), while P5β stops sharing its $H^+$ and P6 becomes protonated (both displaying fairly negative $\Delta\delta_P$ values). P3 is also shielded in the process. Apart from the influence of the ligand conformational change, this is due to the fact that it forms a strong H-bond with P2. Conversely, P4 is deshielded during the protonation step, since after the conformational flip it ends up linked to two $K^+$ ions (instead of one $K^+$ and a $H^+$ in $[MgK_3(H_2L)]^{7-}$). To verify the structural model proposed for $[MgK_3(H_3L)]^{6-}$, we calculated the change in the chemical

shifts of the signals during the process $H_3L^{11-} \rightarrow [MgK_3(H_3L)]^{6-}$ ($\Delta\delta_c$ in Table S1). P2 and P1$\alpha$ are substantially shielded upon magnesium complexation ($\Delta\delta_c < 0$), due to the fact that they are bound to the $Mg^{2+}$ ion. P1β, however, is only mildly shielded, since it exchanges a $H^+$ for one $Mg^{2+}$ and one $K^+$ ions. P3 ends up being linked to a $K^+$ ion, so it expected to be deshielded. However, this is not the case (small negative $\Delta\delta_c < 0$), which is indicative of the high strength of the P3-P2 H-bond in $[MgK_3(H_3L)]^{6-}$. P5$\alpha$, P5β and P6 signals move upfield, possibly because a proton moves away from P3-P4 and establishes a strong H-bond between P5$\alpha$ and P6, allowing P5β to retain the proton that shares with P6 in $H_3L^{11-}$. P4 is the only nucleus that has a positive $\Delta\delta_c$, since in $[MgK_3(H_3L)]^{6-}$ it does not establish H-bonds and is deshielded by two proximate $K^+$ ions.

Towards acidic media, the next protonated magnesium complex is $[MgK_2(H_4L)]^{6-}$. It is not a particularly stable complex, having a very narrow window of pH where it is abundant (around pH = 7; Figure 6b). Its formation is accompanied by large positive $\Delta\delta_P$ values for P1$\alpha$ and P2. A possible structural model that accounts for this observation involves the approach of both nuclei to potassium ions (see Figure S8). It is probable that this structural change compels P1$\alpha$ to move farther away from the $Mg^{2+}$ cation, being depolarized by the double positive charge, and explaining the large positive value of $\Delta\delta_P$. P3 and P5$\alpha$ are also deshielded, suggesting that the former binds tighter to the $K^+$ ion, while the latter loses the H-bond with P6. During the process $[MgK_3(H_3L)]^{6-} \rightarrow [MgK_2(H_4L)]^{6-}$, P1β, P4 and P5β signals move upfield, because the first one moves away from a potassium ion, there is a $K^+/H^+$ exchange around the second nucleus, and the third phosphate group establishes a new H-bond with P6. Table S1 lists the $\Delta\delta_c$ values, calculated from $[K_3(H_4L)]^{7-}$ to $[MgK_2(H_4L)]^{6-}$. P2 and P5β exhibit the most negative $\Delta\delta_c$ values, in line with the fact that P2 binds the entering $Mg^{2+}$ ion, while P5β suffers a $K^+/H^+$ exchange in its surroundings. P1β and P4 also have $\Delta\delta_c < 0$, since the first one starts to coordinate the $Mg^{2+}$ ion, losing the connection with a $K^+$, while the second probably reinforces its H-bond with P3, ended up being protonated. On the contrary, P1$\alpha$ is deshielded during the process $[K_3(H_4L)]^{7-} \rightarrow [MgK_2(H_4L)]^{6-}$, which indicates a strong through-space depolarization effect by the $Mg^{2+}$ cation.

The protonation of $[MgK_2(H_4L)]^{6-}$ leads to a highly stable complex, $[MgK(H_5L)]^{6-}$, abundant between pH = 5 and pH = 7 (Figure 6b). During this process, P1$\alpha$, P1β and P2 chemical shifts decrease noticeably ($\Delta\delta_P < 0$). A structural model for $[MgK(H_5L)]^{6-}$ compatible with this observation is shown in Figure S8. P1$\alpha$ and P2 are no longer connected to a potassium ion, reinforcing the $Mg^{2+}$-P2 coordinative bond, whereas P1β establishes a new H-bond with P6. P3 loses the bond with a $K^+$ ion in $[MgK(H_5L)]^{6-}$ ($\Delta\delta_P < 0$), but the surrounding of P4 does not change substantially (it keeps connected to a $H^+$ and $K^+$ ions; $\Delta\delta_P \approx 0$). P5$\alpha$, P5β and P6 signals move upfield, because in the process $[MgK_2(H_4L)]^{6-} \rightarrow [MgK(H_5L)]^{6-}$ P5$\alpha$ set up a new strong intrapyrophosphate H-bond with P5β, while P6 is no longer bound to a potassium cation. Regarding the $\Delta\delta_c$ values in Table S1, these were determined for the reaction $[K_2(H_5L)]^{7-} \rightarrow [MgK(H_5L)]^{6-}$. In this process, P1$\alpha$'s chemical environment does not change significantly ($\Delta\delta_c \approx 0$), while P1β is slightly deshielded, since it becomes deprotonated and, probably, the P1β-P6 H-bond leads P1β to be less involved in the $Mg^{2+}$ coordination. P2 is highly shielded, possibly due to the fact that it is bound to the magnesium ion instead of a $K^+$ cation. P3 and P4 signals move upfield as well ($\Delta\delta_c < 0$). This phenomenon is not easy to explain. Apart from the fact that other microspecies may be coexisting in solution, it is feasible that P3 H-bonds are stronger than the analogous bonds in $[K_2(H_5L)]^{7-}$, whereas, in the case of P4, the shielding contribution of its protonation may predominate over the deshielding effect of binding to a $K^+$ ion. When going from $[K_2(H_5L)]^{7-}$ to $[MgK(H_5L)]^{6-}$, P5$\alpha$, P5β and P6 have negative $\Delta\delta_c$ values. In the reaction, P5$\alpha$ establishes a new strong H-bond with P5β, while the latter and P6 are no longer linked to a potassium ion. Moreover, P6 becomes protonated in $[MgK(H_5L)]^{6-}$.

Finally, below pH 6, the protonation of $[MgK(H_5L)]^{6-}$ produces $[MgK(H_6L)]^{5-}$. During this step, P1$\alpha$ and P1β are shielded to a great extent ($\Delta\delta_P < 0$). A possible protonation and complexation scheme of $[MgK(H_6L)]^{5-}$ is shown in Figure S8, where P1$\alpha$ starts to coordinate the magnesium metal ion, while P1β is now protonated. P2, however, is slightly

deshielded in the same process, probably because it remains coordinated to the magnesium ion and keeps sharing one $H^+$. The proton initially located between P2 and P3 in $[MgK(H_5L)]^{6-}$ was transferred to P3, giving an explanation of its negative $\Delta\delta_P$. P4, though, does not suffer any significant change in its chemical environment ($\Delta\delta_P \approx 0$). In the process $[MgK(H_5L)]^{6-} \rightarrow [MgK(H_6L)]^{5-}$, P5$\alpha$ exhibit a medium negative $\Delta\delta_P$, but it remains bound to a $K^+$ and a $H^+$ ion. This is suggestive of the formation of a very strong H-bond with P6, which also accounts for the fact that $\Delta\delta_P < 0$ for the latter. In the case of P5β, the NMR chemical shift move upfield substantially, possibly because it no longer shares any of its $H^+$ ions. To validate this structural model, we calculated the change in the chemical shifts of the peaks during the process $[K(H_6L)]^{7-} \rightarrow [MgK(H_6L)]^{5-}$ (see $\Delta\delta_c$ values in Table S1). P1$\alpha$ and P1β are shielded to a significant extent, due to the fact that they are involved in the magnesium coordination. P2, however, is not greatly shielded, since it undergoes a $H^+/Mg^{2+}$ exchange. In the same step, P5$\alpha$ starts to share a proton with P6, while the latter and P5β are no longer linked to a $K^+$ cation (the three nuclei have $\Delta\delta_c < 0$). In any case, there may be other microspecies of $[MgK(H_6L)]^{5-}$ in solution with different protonation and complexation patterns, whose structures can account for the negative $\Delta\delta_c$ values registered for P3 and P4.

### *2.3. Computational Modeling*

Figure 6d shows the optimized geometries of the $K^+$-$Mg^{2+}$-Ins$P_8$ complex predominant at physiological pH (RB3LYP/3–21+G*/LANL2DZ SMD level of theory). In general terms, it is in line with the structural model proposed from the NMR data.

## References

26. Veiga, N.; Torres, J.; MacHo, I.; Gómez, K.; González, G.; Kremer, C. Coordination, Microprotonation Equilibria and Conformational Changes of Myo-Inositol Hexakisphosphate with Pertinence to Its Biological Function. Dalt. Trans. 2014, 43, 16238–16251. https://doi.org/10.1039/c4dt01350f.

28. Hager, A.; Wu, M.; Wang, H.; Brown, N.W.J.; Shears, S.B.; Veiga, N.; Fiedler, D. Cellular Cations Control Conformational Switching of Inositol Pyrophosphate Analogues. Chemistry 2016, 22, 12406–12414. https://doi.org/10.1002/chem.201601754.

36. Frassineti, C.; Ghelli, S. NMR as a Tool for Determining Protonation Constants of Nutral Polyprotic Bases in Solution. Anal. Biochem. 1995, 231, 374–382.

45. Veiga, N.; Torres, J.; Macho, I.; Gómez, K.; Godage, H.Y.; Riley, A.M.; Potter, B.V.L.; González, G.; Kremer, C. Inframolecular Acid–Base and Coordination Properties towards Na+ and Mg2+ of Myo-Inositol 1,3,4,5,6-Pentakisphosphate: A Structural Approach to Biologically Relevant Species. J. Chem. Soc. Dalt. Trans. 2013, 42, 6021–6032. https://doi.org/10.1039/c2dt31807e.

46. Torres, J.; Domínguez, S.; Cerdá, M.F.; Obal, G.; Mederos, A.; Irvine, R.F.; Díaz, A.; Kremer, C. Solution Behaviour of Myo-Inositol Hexakisphosphate in the Presence of Multivalent Cations. Prediction of a Neutral Pentamagnesium Species under Cytosolic/Nuclear Conditions. J. Inorg. Biochem. 2005, 99, 828–840. https://doi.org/10.1016/j.jinorgbio.2004.12.011.

58. Moedritzer, K. PH Dependence of Phosphorus-31 Chemical Shifts and Coupling Constants of Some Oxyacids of Phosphorus. Inorg. Chem. 1967, 6, 936–939. https://doi.org/10.1021/ic50051a017.
